# Relation of Bicuspid Aortic Valve Morphology to the Dilatation Pattern of the Proximal Aorta: Focus on the Transvalvular Flow

**DOI:** 10.1155/2012/478259

**Published:** 2012-07-29

**Authors:** Evaldas Girdauskas, Kushtrim Disha, Michael-Andrew Borger, Thomas Kuntze

**Affiliations:** ^1^Department of Cardiac Surgery, Central Clinic Bad Berka, Robert-Koch-Allee 9, 99437 Bad Berka, Germany; ^2^Department of Cardiac Surgery, Heart Center Leipzig, Strümpellstr. 39, 04289 Leipzig, Germany

## Abstract

Whether the dilatation of proximal aorta in patients with bicuspid aortic valve is secondary to hemodynamic effects related to the abnormal aortic valve or a primary manifestation of the genetic disorder remains controversial. We discuss in this paper the recent data on the BAV function and transvalvular flow patterns in relation with the dilatation type of the proximal aorta. Different morphological forms of bicuspid aortic valve in relation with the specific transvalvular blood flow patterns are focus of the first paragraph of this paper. In the second part of this paper we present the pathogenetic insight into the different clinically observed phenotypes of bicuspid aortic valve disease (i.e., association of proximal aortic shapes with the specific cusp fusion patterns), based on the data from recent rheological studies.

## 1. Introduction

It is widely accepted that bicuspid aortic valve (BAV) is a very heterogeneous disease and that the different phenotypes of BAV-associated aortopathy may be caused by unique pathogenetic mechanisms [1, 2]. In persons with BAV, the dimensions of the proximal aorta are significantly larger than those in persons with tricuspid aortic valve, even in the absence of significant valvular hemodynamic disturbance [3]. Whether the dilatation of proximal aorta in patients with BAV is secondary to hemodynamic effects related to the abnormal aortic valve or a primary manifestation of the genetic disorder remains controversial. The clarification of this phenomenon is not simply theoretical in nature, since this may significantly affect the surgical approach to the dilated ascending aorta in patients presenting with BAV disease. The prevailing theory of BAV-associated aortopathy will undoubtedly tailor the surgical treatment of this common clinical entity. Moreover, specific BAV phenotypes may require different therapeutic approaches and would support an individual treatment strategy of BAV disease.

 In the face of the growing body of evidence on BAV function, we found it important to address the issue of systolic transvalvular flow through the BAV. Indeed, this is one of the crucial points which may resolve the controversy regarding the pathogenesis of BAV-associated aortopathy. We discuss in this paper the recent data on the BAV function and transvalvular flow patterns in relation with the dilatation type of the proximal aorta. These rheological studies are supportive of hemodynamic origin of the distinct BAV phenotypes and will be discussed in details in the following paragraphs.

## 2. BAV Morphology and Transvalvular Flow

**Figure 1:**
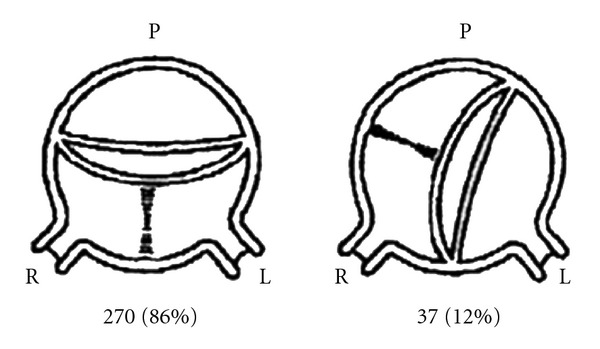
Cusp fusion patterns in BAV disease [4].

The variable morphology of BAV is a result of different cusp fusion patterns, as demonstrated by a large pathologic study from Mayo Clinic, Rochester [4]. The two most common patterns of cusp fusion in BAV disease are fusion of the left and right coronary cusp, which occurs in 70–85% of cases and fusion of the right and noncoronary cusp, which is less common and occurs in the remaining 15–30% of BAV cases (Figure 1).

A more detailed classification of distinct BAV morphological variants has been published by Sievers and Schmidtke [5]. This classification system respects the number of raphes, the spatial position of valve cusps and raphes, and the functional status of the valve.

**Figure 2:**
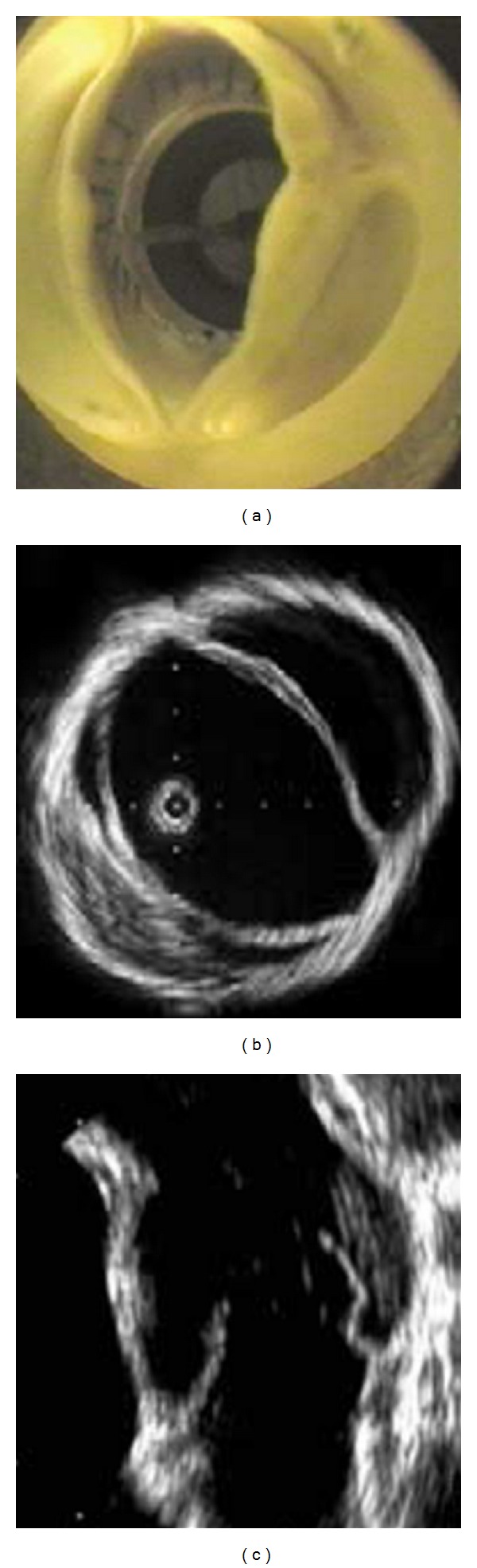
Asymmetric opening of BAV during systole [6].

The asymmetric opening of even “clinically normal” bicuspid aortic valve has been demonstrated experimentally by Dr. Robicsek and coauthors in their pioneering in vitro study [6] (Figure 2).

The orifice of the open BAV has been shown to be irregular and dome shaped, which is caused by the restricted mobility of conjoined leaflet. This asymmetric and morphologically stenotic orifice results in a nonaxial, turbulent transvalvular flow jet, which propagates eccentrically towards the wall of ascending aorta [6]. The authors hypothesized that this uneven wall stress distribution in the proximal aorta may promote the development of proximal aortic dilatation. A novel parameter “cusp opening angle” (i.e., degree of valve leaflet alignment to the outflow axis in systole) was introduced recently by Della Corte and coauthors to quantify cusp motility in the setting of right-left fusion type of bicuspid aortic valves [7]. The latter authors were able to demonstrate a prognostic correlation of this parameter with yearly rate of aortic growth in a multivariable analysis. Systolic flow deflection towards the right, which was associated with inhomogeneous wall stress distribution in the proximal aorta has been demonstrated in the setting of right-left fusion type of BAV [7]. Moreover, a significant correlation has been recently demonstrated between the degree of eccentricity of the systolic transvalvular flow and the severity of the proximal aortic dilatation in the pediatric BAV population (i.e., the larger the angle of misdirected flow with the aortic outflow axis, the larger the proximal aortic diameter) [8]. Barker and coauthors evaluated wall shear stress (WSS) in the ascending aorta of BAV patients using phase-contrast MRI [9]. They introduced a novel parameter of “shear range index,” which measures the shear symmetry along the lumen circumference. These authors demonstrated convincingly that the spatial distribution and magnitude of systolic WSS in BAV patients was significantly different from TAV patients [9]. Moreover, the shear range index varied significantly among the BAV patients, which supports the heterogeneous pattern of aortic dilatation in BAV disease.

**Figure 3:**
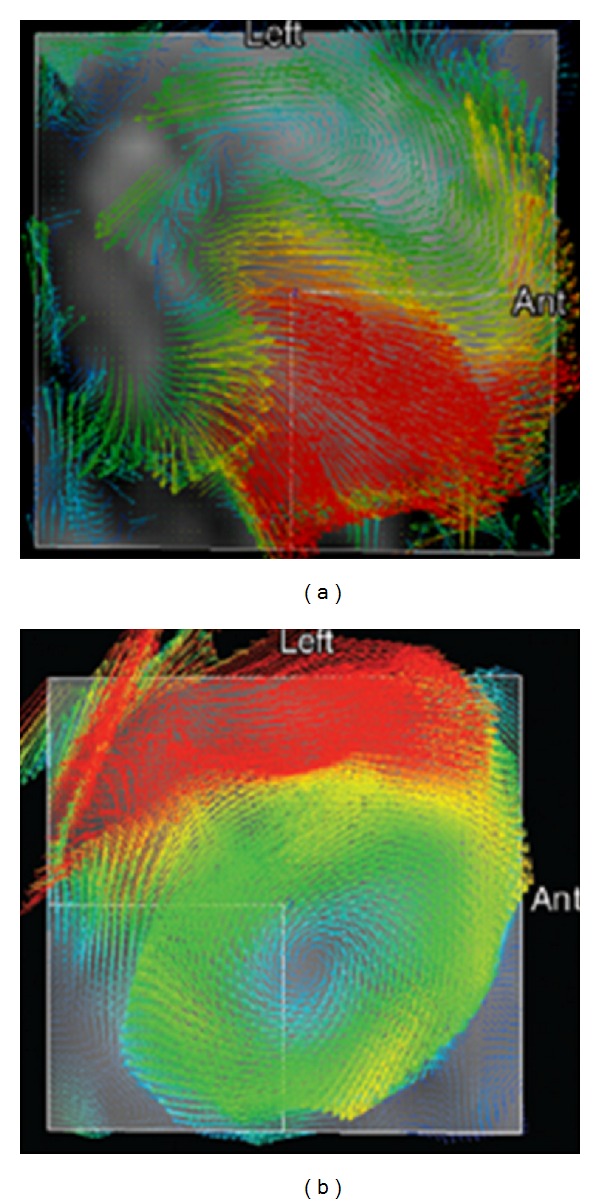
Unique transvalvular flow patterns in BAV patients with right-left cusp fusion (a) and right-noncoronary cusp fusion (b) [10].

The most important contribution in this context, which analyzed the transvalvular blood flow patterns in BAV patients using sophisticated four-dimensional magnetic resonance imaging, was published by Hope and coauthors [10]. These investigators demonstrated a nested helical systolic flow in the ascending aorta in patients with BAV including those without ascending aortic aneurysm or aortic valve stenosis. This strongly suggests that the abnormal systolic flow pattern is not secondarily to a dilated aorta or to aortic valve dysfunction and may be implicated in the pathogenesis of BAV aortopathy. Importantly, the authors found specifically two systolic transvalvular flow patterns in patients with BAV, which strongly correlated with the two most common cusp fusion types [10]. The fusion of the right-left coronary cusps was associated with a right-anteriorly directed systolic flow jet with a marked peripheral skewing towards the convexity of the ascending aorta (Figure 3(a)). BAV patients with the less common right-noncoronary cusp fusion demonstrated a left posteriorly directed eccentric flow with propagation towards the proximal aortic arch (Figure 3(b)).

These data demonstrated clearly that different morphologic forms of BAV may generate specifically oriented systolic flow jets in the proximal aorta. As a consequence of this, it may be assumed that different systolic flow patterns may result in specific segments of aortic aneurysm formation in BAV patients. Specifically, fusion of right-left cusps, which produces a right anteriorly oriented flow jet may explain the larger aortic root dimensions and asymmetric dilatation of midascending tract, commonly seen in these BAV patients [11]. Inversely, fusion of the right-noncoronary cusps gives an origin to left posteriorly directed flow-jet, which propagates further towards the proximal aortic arch and may result in increased aortic arch dimensions in this subgroup of BAV patients [12].

Most recent in vitro studies provide some valuable insights into the transvalvular hemodynamics of BAV patients. Nathan and coauthors used finite element analysis in order to investigate the wall stress in the proximal aorta of BAV patients [13]. These authors were able to demonstrate significantly increased 99th percentile wall stress in the BAV group versus in the TAV group [13]. Saikrishnan and colleagues addressed BAV hemodynamics in an in vitro system using a particle image velocimetry [14]. The authors demonstrated an eccentric systolic jet in stenotic BAV, which impinged on the aortic wall on the nonfused cusp side, causing a strong vortex in the nonfused cusp sinus. These findings correlate well with the in vivo flow patterns presented by Hope and coauthors [10]. Moreover, the values of turbulent kinetic energy and shear stress in BAV models were almost twice as large as comparable values in TAV model in the aforementioned study by Saikrishnan and coauthors [14]. Vergara and associates performed most recently a parametric study, based on the simulations of ascending aorta hemodynamics with different configurations of BAV orifice area and valve orientation [15]. Their results showed that aortic wall shear stress was more pronounced in subjects with BAV morphologies, including the nonstenotic cases, as compared with TAV morphology. Moreover, the asymmetry of blood flow was found to be larger for a decreasing BAV area and for a laterolateral BAV configuration [15]. This is in accordance with the previously presented clinical studies on association between BAV fusion types and different proximal aortic aneurysm morphology [11, 12].

 These hemodynamic in vitro and in vivo data provide a pathogenetic support for the clinically observed BAV phenotypes (i.e., association of proximal aortic shapes with the cusp fusion patterns in BAV disease) which will be elucidated in the following paragraph.

## 3. Cusp Fusion Pattern and Proximal Aortic Shape in BAV Disease

There is emerging evidence from the recent literature that different cusp fusion patterns in BAV disease are associated with specific dilatation patterns of the proximal aorta. This clinically observed linkage between bicuspid valve morphology and the lesion of proximal aorta has led to phenotypic classification of BAV disease which incorporates both valve and proximal aortic anatomy [1, 2, 11].

**Figure 4:**
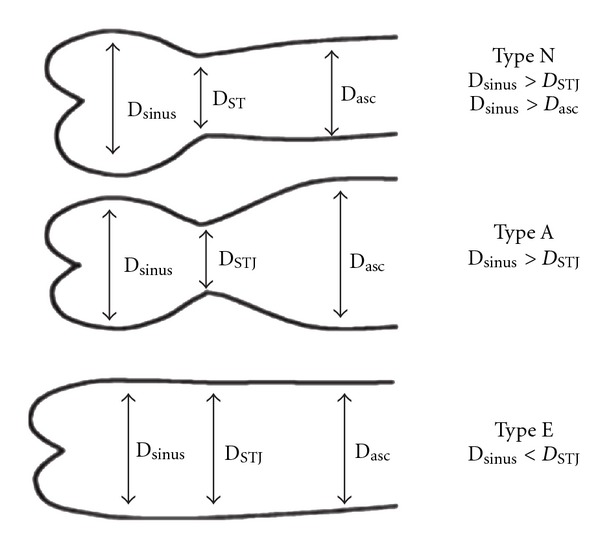
Three proximal aortic shapes in BAV disease [2].

Russo and coworkers were able to demonstrate that BAV patients with right-left cusps fusion had a significantly larger aortic root diameter and were significantly younger at the time of surgery versus BAV patients with right-noncoronary cusps fusion [11]. Moreover, histological changes of ascending aortic wall were more pronounced in patients with right-left cusps fusion versus right-noncoronary cusps fusion. The degree of aortic wall degeneration, expressed as prevalence of fibrosis, cystic medial necrosis, elastic fragmentation, and inflammation has been shown to be significantly higher in BAV patients with fusion of the right and left coronary cusps. Echocardiographic examination of BAV patients with different cusps fusion patterns brought similar findings: right-left cusps fusion was associated with larger aortic root diameter, whereas the fusion of right-noncoronary cusps was associated with larger aortic arch diameter [16]. Moreover, right-left cusps fusion correlated with a higher aortic stiffness index and lower distensibility at the level of aortic root. These authors hypothesized that the differences in spatial propagation of blood flow in different morphologic BAV forms may lead to an inhomogeneous distribution of wall stress and, consequently, to specific patterns of aortopathy. In their subsequent study, Schaefer and coworkers were able to identify three specific proximal aortic shapes in BAV disease (Figure 4) [2].

Based on analysis of echocardiographic data, right-left cusps fusion was associated with normal aortic shape (i.e., Type N) but larger diameters of aortic root [2]. In contrast, right-noncoronary cusps fusion correlated with larger distal ascending aorta and aortic arch dimensions (i.e., Type A). Type E aortic shape illustrates one specific form of BAV disease, so-called “root phenotype.” This is a relatively rare form of BAV disease (10–15% of BAV patients), which affects predominantly young male patients and is associated with aortic valve insufficiency due to aortic annular dilatation. Root phenotype may represent the predominantly genetic form of BAV disease, which is less influenced by the hemodynamic factors. Similar findings brought another retrospective echocardiographic study by Holmes and colleagues [17]. On the contrary, Thanassoulis and coauthors found in their echocardiographic study a close relationship between the right-left cusps fusion and increased risk of rapid aortic dilatation in BAV patients [18]. Possible explanations for these differences between the findings of those two studies might be the specific definition of aortic dilatation (i.e., aortic measurements converted to *z* scores on the basis of body surface area [17] versus the parameter of rapid aortic dilatation [18]) and the apparently different levels of measurement of aortic diameters (i.e., specific measurement of ascending aorta above the sinotubular junction at the point of maximum diameter versus measurements of the whole aortic root).

## 4. Functional Status of BAV and Proximal Aortic Shape

**Figure 5:**
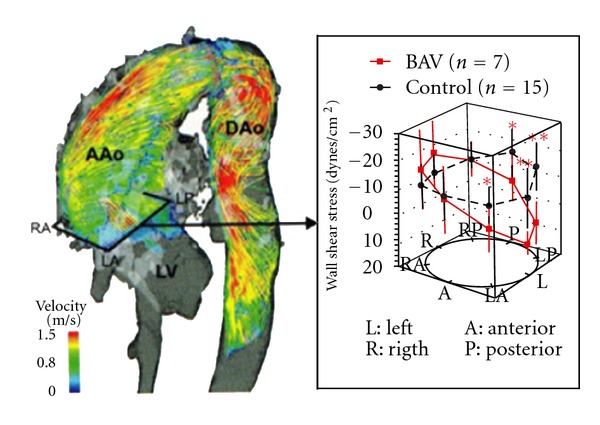
Differences in aortic wall shear stress in BAV patients versus TAV controls [19].

The strong correlation between the functional status of BAV (i.e., valve stenosis) and the dilatation pattern of the proximal aorta has been demonstrated by Cotrufo and Della Corte [1]. These authors identified “BAD MATE” syndrome in order to describe the common association between BAV stenosis and asymmetric dilatation of the tubular ascending aorta, starting from the sinotubular junction and involving the convexity (i.e., the greater curvature) of the vessel. This association has been proposed to be of pathogenetic origin and result of eccentric transvalvular flow jet through stenotic BAV, which causes asymmetrical wall stress distribution in the proximal aorta. The asymmetric wall shear stress distribution in BAV patients leading to the flow-induced vascular remodeling was demonstrated recently in multiple models (Figure 5) [19].

Consistently with these hemodynamic findings, asymmetric histological pattern of BAV aortopathy has been demonstrated by a series of consecutive biomolecular investigations by Cotrufo and coworkers. These authors were able to convincingly show in several studies an asymmetric spatial pattern of extracellular matrix protein expression and smooth muscle cell changes in the convexity versus concavity of BAV aorta [20] in bicuspid versus tricuspid aortic valve stenosis [21] and in BAV versus Marfan's syndrome [22].

In summary, there is a growing amount of evidence that bicuspid aortic valve morphology with resulting eccentric transvalvular blood flow may have a major impact on the proximal aortic shape in BAV disease. Data from the recent studies require a thorough reevaluation of the ongoing controversy regarding the origin of BAV aortopathy [23]. The clarification of this phenomenon is not simply theoretical in nature, since this may significantly affect the surgical approach to the ascending aorta in patients presenting with BAV disease. Given the apparent heterogeneity of BAV disease, there is an urgent need for improvement of methods and criteria of both diagnosis and treatment of different forms of BAV aortopathy. From the practical point of view, there is a demand for new diagnostic methods to distinguish the more from the less “malignant” forms of BAV aortopathy. Such a risk stratification tool may include some combinations of protein assays (e.g., metalloproteinase 2 levels) and sophisticated MRI analyses [8], in order to predict the clinical risk of adverse aortic events. The long-term scope should be the development of new management algorithms and recommendations, in order to better comply with the multiplicity of phenotypes and provide the best tailored approach.
